# *Mycoplasma genitalium* incidence, persistence, concordance between partners and progression: systematic review and meta-analysis

**DOI:** 10.1136/sextrans-2018-053823

**Published:** 2019-05-04

**Authors:** Manuel Cina, Lukas Baumann, Dianne Egli-Gany, Florian S Halbeisen, Hammad Ali, Pippa Scott, Nicola Low

**Affiliations:** 1 Institute of Social and Preventive Medicine, University of Bern, Bern, Switzerland; 2 The Kirby Institute, University of New South Wales, Sydney, New South Wales, Australia; 3 Department of Pathology and Biomedical Science, University of Otago, Christchurch, New Zealand

**Keywords:** mycoplasma, infectious diseases, systematic reviews, meta-analysis

## Abstract

**Background:**

*Mycoplasma genitalium* is increasingly seen as an emerging sexually transmitted pathogen, and has been likened to *Chlamydia trachomatis*, but its natural history is poorly understood. The objectives of this systematic review were to determine *M. genitalium* incidence, persistence, concordance between sexual partners and the risk of pelvic inflammatory disease (PID).

**Methods:**

We searched Medline, EMBASE, LILACS, IndMed and African Index Medicus from 1 January 1981 until 17 March 2018. Two independent researchers screened studies for inclusion and extracted data. We examined results in forest plots, assessed heterogeneity and conducted meta-analysis where appropriate. Risk of bias was assessed for all studies.

**Results:**

We screened 4634 records and included 18 studies; six (4201 women) reported on incidence, five (636 women) on persistence, 10 (1346 women and men) on concordance and three (5139 women) on PID. Incidence in women in two very highly developed countries was 1.07 per 100 person-years (95% CI 0.61 to 1.53, I^2^ 0%). Median persistence of *M. genitalium* was estimated from one to three months in four studies but 15 months in one study. In 10 studies measuring *M. genitalium* infection status in couples, 39%–50% of male or female sexual partners of infected participants also had *M. genitalium* detected. In prospective studies, PID incidence was higher in women with *M. genitalium* than those without (risk ratio 1.73, 95% CI 0.92 to 3.28, I^2^ 0%, two studies).

**Discussion:**

Incidence of *M. genitalium* in very highly developed countries is similar to that for *C. trachomatis*, but concordance might be lower. Taken together with other evidence about age distribution and antimicrobial resistance in the two infections, *M. genitalium* is not the new chlamydia. Synthesised data about prevalence, incidence and persistence of *M. genitalium* infection are inconsistent. These findings can be used for mathematical modelling to investigate the dynamics of *M. genitalium*.

**Registration numbers:**

CRD42015020420, CRD42015020405

## Introduction

*Mycoplasma genitalium* is increasingly seen as an emerging sexually transmitted pathogen.^1–4^ *M. genitalium* is a cause of non-gonococcal urethritis^1^ and cervicitis,^3^ and associations with pelvic inflammatory disease (PID), other reproductive tract complications in women and adverse pregnancy outcomes have been found.^3^ *M. genitalium* has thus been called the ‘new chlamydia’.^5^ In a previous systematic review, we found a prevalence of *M. genitalium* of approximately 1% in sexually active heterosexuals in the general population, which is similar to that reported for *Chlamydia trachomatis* aged 16–44 years.^6^ In sex workers, men who have sex with men (MSM) and clinic-based populations prevalence was higher and more variable.^7^ The increasing availability of nucleic acid amplification tests (NAAT) that detect *M. genitalium* has resulted in debate about the need for widespread testing of asymptomatic populations.^8–10^ But increased testing and treatment are likely to increase the already high proportion of antimicrobial resistant *M. genitalium* since resistance to macrolides often emerges during treatment.^11 12^

Mathematical modelling could help to understand the balance of benefits and harms that widespread testing and treatment interventions bring.^9^ To develop mathematical models, we need robust estimates from clinical and epidemiological studies about key variables that determine the spread of infection in a population^13^ and progression to complications.^9^ These variables include the incidence of infection; persistence of untreated infection, which can be used to estimate the duration of infectiousness;^14^ concordant *M. genitalium* status between sexual partners, which can be used to derive the transmission probability;^15 16^ and the probability that *M. genitalium* in the lower genital tract ascends to cause PID, which can result in tubal factor infertility and ectopic pregnancy.^17^ In the first published model of *M. genitalium* transmission and the impact of screening, the authors noted the uncertainty about the values used for parameters describing the natural history of infection and progression to PID.^9^ The objectives of this study were to systematically review the research literature to estimate the incidence of *M. genitalium* infection, persistence of untreated *M. genitalium*, concordance of *M. genitalium* detection and the risk of developing PID.

## Methods

This systematic review is one of two linked reviews that used a single search strategy and are described in two protocols.^18 19^ A review of the prevalence of *M. genitalium* has been published.^7^ We report our findings according to the Preferred Reporting Items for Systematic Reviews and Meta-Analyses (PRISMA, online supplementary file 1).^20^

10.1136/sextrans-2018-053823.supp1 Supplementary data

### Eligibility criteria

We included studies of *M. genitalium* detected by NAAT. Study populations were women and men older than 13 years in any country. Eligible study designs were as follows: for incidence of *M. genitalium*, cohort studies with participants who were uninfected at baseline; for persistence, cohort studies that followed people with untreated *M. genitalium* infection; for concordance, cross-sectional studies which enrolled couples or sexual partners of index cases, excluding studies in which the infection status of a sexual partner was based on self-report; for incidence of PID, cohort or nested case–control studies that compared women with and without *M. genitalium*, excluding cross-sectional studies and case–control studies in which it could not be established that *M. genitalium* infection preceded a diagnosis of PID.

### Information sources and search strategy

We searched Medline and EMBASE databases for publications in any language from 1 January 1981 until 12 July 2016 and updated the search to 17 March 2018. We used thesaurus headings and free-text terms that combined *Mycoplasma* or *Mycoplasma genitalium* with genital tract complications (online supplementary file 2, online supplementary text S1–3). We also searched the African Index Medicus, IndMED and LILACS, using the term *Mycoplasma genitalium*. Records were managed using EndNote (V.X8.1; Clarivate Analytics, Philadelphia, Pennsylvania, USA).

10.1136/sextrans-2018-053823.supp2 Supplementary data

### Study selection

Abstracts published before 1 January 1991 were excluded. Two reviewers (MC, LB) assessed study eligibility independently, using a pre-piloted screening form. We resolved differences by discussion or adjudication by a third reviewer (NL).

### Data collection

Two reviewers (MC, LB, DE-G, HA) extracted data independently. Differences were resolved by discussion or adjudication. We extracted data using a standardised, piloted data extraction form in a Research Electronic Data Capture database (REDCap; Vanderbilt University, Nashville, Tennessee, USA). For each study, we extracted data about study characteristics, methods and results. For studies reporting persistence of *M. genitalium* only in graphs, we used Plot Digitizer software^21^ to record numerical data. We labelled studies with the country in which the data were collected and added consecutive numbers for studies subsequently identified from the same country. Studies reported in the linked systematic review of *M. genitalium* prevalence have the same study identifier (online supplementary table S1).^7^ We contacted authors to clarify details of study methods and results, where necessary.

### Risk of bias in individual studies

For cohort or nested case–control studies, we adapted a tool published by the Cochrane Bias Methods Group.^22^ For cross-sectional studies, we applied a previously used checklist^7 23^ (online supplementary file 2, online supplementary text S4).

### Summary measures

We defined incidence in cohort studies as the rate of new *M. genitalium* infections per 100 person-years of observation in individuals with a negative *M. genitalium* test, either at baseline or a negative test of cure following treatment of a prevalent infection. We defined persistence of *M. genitalium* infection in cohort studies as the proportion of study participants at each follow-up visit with a positive test result. We assessed concordance of *M. genitalium* infection status in cross-sectional studies as the proportion of sexual partners of an infected index case that had a positive test result. We assessed the development of PID in cohort studies and calculated the odds ratio (OR) or risk ratio (RR) with 95% confidence intervals (CI) for PID in participants with and without *M. genitalium* infection at baseline.

### Synthesis of results

We used Stata (V.13.1; StataCorp, College Station, Texas, USA) for statistical analysis. We examined data about incidence, concordance and PID in forest plots. We stratified studies reporting incidence according to the level of development of the country in which the study was conducted, categorised as very high, high, medium and low using the United Nations Development Programme Human Development Index (HDI),^24^ as we found differences between countries with higher or lower human development index in our linked review of *M. genitalium* prevalence.^7^ We stratified studies reporting concordance according to study design: studies can enrol couples irrespective of infection status and test all individuals for *M. genitalium* (referred to as partner studies), or can enrol an index case with *M. genitalium* and then test their partners (referred to as index case studies). We calculated the percentage (with 95% CI) concordance separately for women and men. We assessed the percentage of study variability between studies caused by heterogeneity other than that due to chance with the I^2^ statistic.^25^ Meta-analysis was conducted when deemed appropriate using fixed or random effects models. For estimates of incidence, we estimated a summary estimate of the incidence rate per 100 person-years of follow-up (with 95% CI). For concordance, we applied the Freeman-Tukey arcsine transformation to the proportions before meta-analysis and back transformed the summary estimate and its 95% CI.

The data about persistence of *M. genitalium* are presented graphically (Excel:mac 2008, V.12.3.6; Microsoft Corporation, Redmond, Washington, USA) without statistical analysis because we anticipated that the results would be too heterogeneous to combine.^16^ We conducted a subgroup analysis, using a test of interaction, of differences in concordance by study design.

### Risk of bias across studies

We did not test for small study biases with funnel plots because of the small number of included studies.

## Results

We identified 4634 records and, after exclusion of duplicates and articles published before 1991, we screened 3820 records. We included 18 studies, some of which reported on more than one review question (table 1, online supplementary file 2, figure S1, table S1). Six studies reported on incidence,^5 26–30^ five reported on persistence,^5 26–28 31^ 10 reported on concordance between partners^31–40^ and three studies reported on development of PID.^5 41 42^

**Table 1:** Included studies (n=18), ordered according to outcomes reported

Study identifier	Study population	Study design	Review topics
Great Britain 2^5^	Female students aged ≤27 years; universities and further education colleges, London, Great Britain	Cohort study	Incidence, persistence, PID
Kenya 2^26^	Female sex workers aged 18–35 years; Kariobangi Nairobi City Council, Nairobi, Kenya	Cohort study	Incidence, persistence
Kenya 3^28^	Female sex workers, median age 35.3 years; municipal STI clinic Mombasa, Kenya	Cohort study	Incidence, persistence
Uganda 1^27^	Female sex workers aged 18–40 years; red light areas within southern Kampala, Uganda	Cohort study	Incidence, persistence
Australia 3^29^	Young women aged 16–25 years; primary health clinics in Melbourne, Australia	Cohort study	Incidence
USA/Kenya 1^30^	High-risk women aged 18–45 years; research clinics in Mombasa and Nairobi, Kenya and Birmingham, USA	Cohort study	Incidence
USA 7^31^	Women aged 14–17 years and their partners; urban primary healthcare centres, Indianapolis, USA	Cohort study, cross-sectional sampling of couples	Persistence, concordance
Great Britain 8^34^	Women and their partners; STI clinic, St. Mary's Hospital, London, Great Britain	Cross-sectional	Concordance
Great Britain 9^33^	Men and their partners; STI clinic, St. Mary's Hospital, London, Great Britain	Cross-sectional	Concordance
Peru 1^35^	Couples, men aged 19–60 years, women aged 18–55 years; two STI clinics, Lima, Peru	Cross-sectional	Concordance
USA 8^32^	Mexican-American and African-American women with non-viral STI aged 14–45 years and their male partners; San Antonio Metropolitan Health District STI Clinic, USA	Cross-sectional	Concordance
Sweden 2^36^	Men aged 16–67 years and their partners; Örebrö University Hospital STI clinic, Sweden	Index cases and sexual partners	Concordance
Sweden 5^37^	Women aged 14–55 years and men aged 17–67 years and their partners; STI clinic, Falun, Sweden	Index cases and sexual partners	Concordance
Sweden 11^38^	Women aged 15–54 years and their partners; Örebrö University Hospital STI clinic, Sweden	Index cases and sexual partners	Concordance
Sweden 12^39^	Male patients with symptomatic recurrent and/or persistent urethritis aged 20–47 years and their partners; STI clinic, Karolinska Hospital, Stockholm, Sweden	Index cases and sexual partners	Concordance
Australia 6^40^	Partners of index cases with *M. genitalium*, median age of female heterosexual partners 26 years; male heterosexual partners 28 years; MSM partners 29 years; Melbourne Sexual Health Centre, Australia	Index cases and sexual partners	Concordance
Sweden 10^41^	Women after medical or surgical termination of pregnancy aged 17–40 years; gynaecological outpatient department Malmö University Hospital, Sweden	Nested case–control study	PID*
USA 6^42^	Women after treatment and cure of PID aged 14–37 years; multiple clinical sites in the USA	Cohort study	PID†

*Post-abortion upper genital tract infection.

†Endometritis considered as confirmed PID.

MSM, men who have sex with men; PID, pelvic inflammatory disease; STI, sexually transmitted infection.

### Incidence

We included six studies (online supplementary table S2),^5 26–30^ with a total of 4201 female participants at baseline and follow-up of 3461 person-years. Two studies were conducted in countries with a very high HDI, with students (Great Britain 2)^5^ and with attendees of primary healthcare clinics (Australia 3).^29^ All three studies from countries with a low HDI were conducted with female sex workers in (Uganda 1, Kenya 2 and Kenya 3).^26–28^ Two of the four research clinics which enrolled participants for the USA/Kenya 1 study^30^ approached only female sex workers too. All women in the Uganda 1 study had a positive test for *M. genitalium* at baseline. Incidence was defined as a positive test result in women who had a preceding negative test result.^27^ All studies were at risk of bias (online supplementary table S3). All studies reported more than 20% loss to follow-up or did not report it.^5 26–30^ Only one (Great Britain 2) compared participants followed up until the end of the studies and participants lost to follow-up.^5^

**Figure 1:**
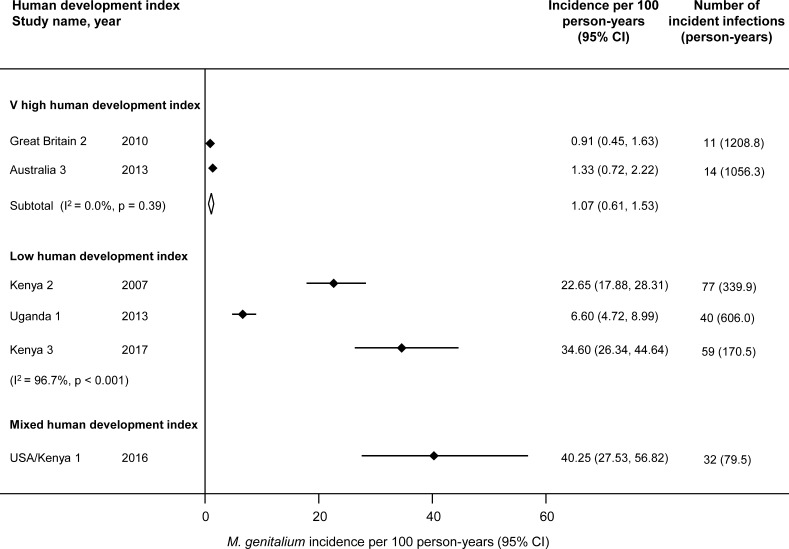
Incident *M. genitalium* infections per 100 person-years by human development index.^24^ Solid diamond and lines show the point estimate and 95% confidence intervals (CI) for each study. The diamond shows the point estimate and 95% CI of the summary estimate. The incidence estimates are plotted on a linear scale.

Figure 1 shows that in countries with a very high HDI, the pooled estimate of incidence was 1.07 per 100 person-years (95% CI 0.61 to 1.53, 2 studies, I^2^ 0%).^5 29^ The incidence rates in studies conducted among female sex workers were higher and too heterogeneous to combine (I^2^ 96.7%).

### Persistent detection of *M. genitalium*

We included five studies,^5 26–28 31^ with a total of 636 female participants at baseline (online supplementary table S4). Three studies were conducted with female sex workers in Kenya and Uganda (Uganda 1, Kenya 2 and Kenya 3).^26–28^ The other two studies were conducted with adolescents enrolled from primary healthcare facilities (USA 7)^31^ and students from educational colleges (Great Britain 2).^5^ Duration of follow-up ranged from 12 weeks in USA 7^31^ to 33 months in Kenya 2.^26^ Specific treatment for *M. genitalium* was not prescribed in any of the studies. All studies were at risk of bias in outcome assessment (online supplementary table S5). In Great Britain 2, women with a positive test result for *C. trachomatis* at baseline received antibiotics if they were in the intervention arm of the underlying randomised control trial but could have been treated before the 12-month follow-up.^5^ In all other studies, participants received either syndromic treatment or treatment for diagnosed *C. trachomatis*, *N. gonorrhoeae* and/or *Trichomonas vaginalis* at one-month to three-month intervals. Two studies (Great Britain 2, Kenya 2) distinguished persistent from re-infections with genotyping.^5 26^

Online supplementary figure S2 shows a rapid decrease in the proportion of women infected in four studies. Median persistence in the three studies of sex workers was one to three months. The Great Britain 2 study only assessed *M. genitalium* persistence at one subsequent time point at which 25.9% of participants were still infected after a median of 16 months.^5^ In USA 7, 31.3% of women remained positive at 8 weeks.^31^

### Concordance

We included 10 cross-sectional studies,^31–40^ all of which were conducted in healthcare facilities (online supplementary table S6). Five partner studies enrolled a total of 869 couples irrespective of infection status^31–35^ and five index case studies^36–40^ enrolled a total of 477 people with *M. genitalium* and 480 sexual partners. Only the Australia 6 study enrolled MSM.^40^ All studies were at risk of bias (online supplementary table S7).^31–40^ The response rate at baseline was only assessed in two studies in which it was below 70% (Great Britain 9 and Peru 1).^33 35^

**Figure 2:**
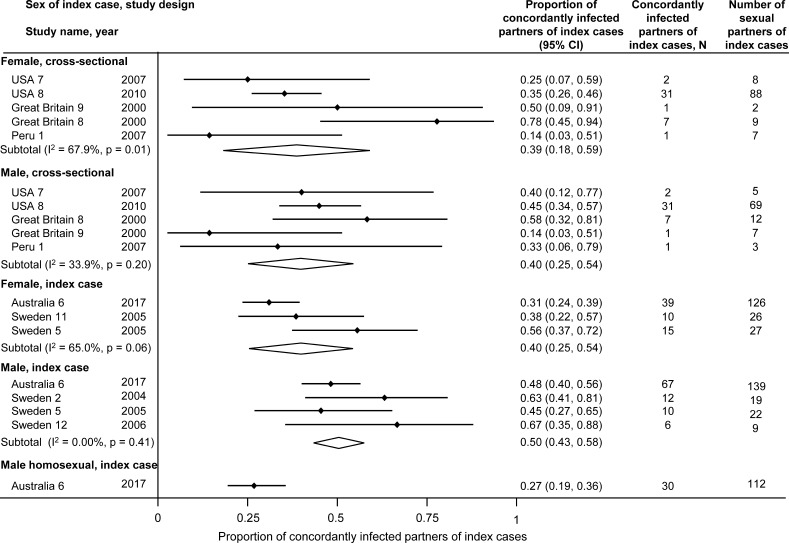
Proportion of concordantly infected sexual partners of individuals with *M. genitalium*, by sex of index case and study design. Solid diamonds and lines show the point estimate and 95% confidence interval (CI) for each study. The diamond shows the point estimate and the 95% CI of the summary estimate. The proportions are plotted on a linear scale.

Figure 2 shows overall concordance rates of 39%–40% among male partners of women with *M. genitalium* and 40%–50% in female partners of infected men, with no marked differences according to study design (online supplementary table S8). Concordance among MSM (Australia 6) was 27% (95% CI 19% to 36%).^40^

### Pelvic inflammatory disease

We included three prospective studies that examined the risk for PID in *M. genitalium*-infected compared with non-infected participants, with a total of 5139 participants at baseline (online supplementary table S9).^5 41 42^ The Great Britain 2 study enrolled female students in London,^5^ USA 6 studied women who had taken part in a randomised controlled trial, after treatment and cure of a first episode of PID^42^ and Sweden 10 was a nested case–control study in women who had undergone medical or surgical termination of pregnancy.^41^ PID was diagnosed by endometrial biopsy in USA 6 and using clinical criteria in Great Britain 2^5^ and Sweden 10.^41^ Follow-up was 6 weeks in Sweden 10,^41^ 12 months in Great Britain 2^5^ and 30 days in USA 6.^42^ All studies were at risk of bias (online supplementary table S10).^5 41 42^ None of the studies assessed whether factors that might be associated with progression to PID were similar between groups or compared individuals followed up with those lost to follow-up.

**Figure 3:**
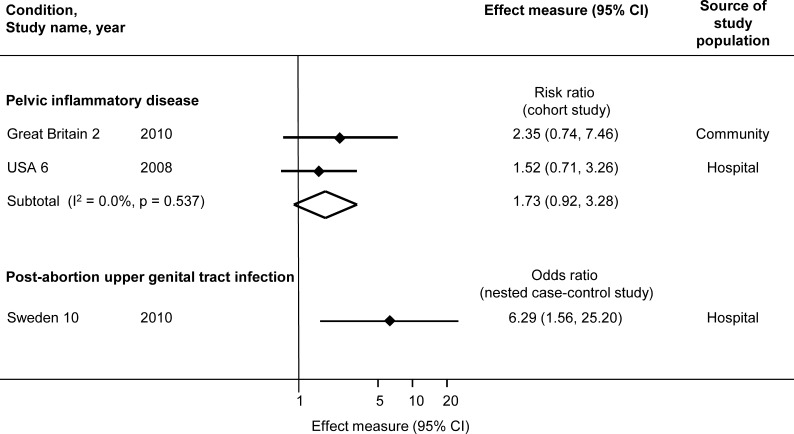
Risk of progression to upper genital tract infection in women with *M. genitalium* compared with women without *M. genitalium*. Solid diamonds and lines show the point estimate and 95% confidence interval (CI) for each study. The open diamond shows the point estimate and the 95% CI of the summary estimate. The effect estimates are plotted on a logarithmic scale.

All studies found an association between *M. genitalium* and PID (figure 3). The summary RR for incident PID in the two cohort studies was 1.73 (95% CI 0.92 to 3.28, I^2^ 0%). The OR for post-abortion upper genital tract infection was 6.29 (95% CI 1.56 to 25.20).^41^

## Discussion

### Main findings

In this systematic review, the incidence of *M. genitalium* was 1.07 per 100 person-years (95% CI 0.61 to 1.53, I^2^ 0%, 2 studies) in women in very highly developed countries. Median duration of persistence of *M. genitalium* was one to three months in four studies but 15 months in one study. In 10 studies measuring *M. genitalium* infection status in heterosexual couples, proportions of concordant results were 39% to 50%. In two prospective studies, the incidence of PID was higher in women with *M. genitalium* than those without (RR 1.73, 95% CI 0.92 to 3.28, I^2^ 0%).

### Strengths and limitations

A strength of our systematic review is the broad search strategy that covered differing topics, which makes it unlikely that we missed important relevant articles. In addition, selection of studies and extraction of data by independent reviewers reduces the risk of errors in data extraction. We assessed the risk of bias in all included studies. The relative importance of the domains of bias affect interpretation depend on the topic. For example, when measuring the duration of persistent detection, accurate assessment of the outcome, untreated infection is important, but most studies were at high risk of bias. The main limitations of the review findings result from the small number of studies overall and between study heterogeneity.

### Interpretation of the findings

Incidence and persistent detection of *M. genitalium*: The findings of this review do not allow an estimate of the duration of *M. genitalium* infectiousness. Estimates based on persistent detection in cohort studies are inconsistent (online supplementary figure S2, online supplementary table S4). Since duration of infection is related to prevalence (assessed in our linked review^7^) and incidence (figure 1), the findings can be compared with this alternative measure (Equation 1): duration of infection=prevalence÷incidence (1)

In this review, three studies estimated all three quantities (online supplementary table S11).^5 26 28^ In Great Britain 2,^5^ the duration of infection, both directly estimated and from Equation 1, was more than one year. In Kenya 2^26^ and Kenya 3,^28^ the duration was less than one year by both methods. In Uganda 1,^27^ the directly estimated duration was less than one year but prevalence was higher than incidence, so Equation 1 results in an estimated duration of more than one year. In two studies that measured incidence and prevalence but not persistent detection, duration of infection could only be obtained using Equation 1, with an estimate of more than one year for Australia 3^29^ and less than one year for USA/Kenya 1^30^ (online supplementary table S11). In all studies, women had opportunities for treatment with antibiotics with some activity against *M. genitalium* at frequencies of as little as a month. The duration of persistent detection was short in all studies that offered treatment every three months or more frequently. With likely inadvertent treatment and re-infection, these cohort studies probably did not measure the persistence of untreated infection. Smieszek and White, who analysed the conflicting findings in the Great Britain 2^5^ and Uganda 1^27^ studies using a mathematical model, favoured a longer duration of infection similar to the Great Britain 2 study.^16^ The uncertainty about the duration of infectiousness of *M. genitalium* contrasts with *C. trachomatis*, for which the literature is extensive and there is broad agreement that prevalence in general populations in high-income countries is around 3%–4%,^23^ incidence is around 4%^43 44^ and average duration of infectiousness is slightly more than one year.^14 45^

*M. genitalium* concordance: The systematic review data suggest some possible differences between *M. genitalium* and *C. trachomatis*. Concordant *M. genitalium* status can be used to estimate the transmission probability of sexually transmitted pathogens.^15 46^ Cross-sectional studies of randomly sampled couples, irrespective of infection status, provide the least biased estimate.^46^ For this reason, we examined concordance separately in partner studies and in index case studies, but actually found similar estimates in both study designs. In cross-sectional studies, *M. genitalium* concordance was 39%–40%. In comparison, *C. trachomatis* concordance in a large cross-sectional study in the USA was 68% (95% CI 56% to 78%) for male partners and 70% (58% to 80%) for female partners.^47^ Findings from our systematic review of *M. genitalium* prevalence suggested that, while overall population prevalence of the two infections is similar, *C. trachomatis* positivity is concentrated in younger age groups.^23^

*M. genitalium* progression to PID: *M. genitalium* was associated with PID in prospective studies (RR 1.73, 95% CI 0.92 to 3.28), with CIs that were compatible with both a small reduction and a substantial increase in risk. The point estimate was slightly lower than that found by Lis *et al*, but their inclusion of cross-sectional studies and studies of post-abortal PID in the same meta-analysis might have overestimated the association.^3^ The increase in risk of PID following *C. trachomatis* is around 1.8 to 2.8.^48 49^ Using data from the Great Britain 2 study and taking into account the low population prevalence of *M. genitalium*, Oakeshott *et al* estimated that the population attributable fraction of PID due to *M. genitalium* was about 4%.^5^

### Implications for research and practice

This review adds to the evidence about the biology, dynamics and natural history of *M. genitalium* as a sexually transmitted pathogen. Additional empirical research is needed to provide robust data about the epidemiology of *M. genitalium* infection in men and to determine the persistence of untreated *M. genitalium* in studies in which inadvertent treatment can be excluded. In the context of evidence of high levels of macrolide resistance in *M. genitalium*,^11 12^ which does not affect *C. trachomatis*, measures for the management and control of these infections are likely to differ. Despite earlier speculation,^5^ the findings of this review, our linked review of prevalence^7^ and evidence about antimicrobial resistance show that *M. genitalium* is not the new chlamydia. The estimates from this systematic review can be used in mathematical modelling studies to investigate differences between the transmission dynamics of *M. genitalium* and *C. trachomatis* and to investigate the potential benefits and harms of control interventions.

**Key messages**

- There is debate about the need for widespread screening for *Mycoplasma genitalium*, but the natural history of this emerging sexually transmitted pathogen is poorly understood.
- *M. genitalium* incidence was 1.07 (95% CI 0.61 to 1.53) per 100 person-years in women in highly developed countries, 39%–50% of infected individuals had a heterosexual partner with *M. genitalium* and the risk ratio for progression to pelvic inflammatory disease was 1.73 (95% CI 0.92 to 3.28).
- The duration of untreated *M. genitalium* infection could not be determined from this review but is probably longer than persistent detection of *M. genitalium*, as measured in most cohort studies, in which inadvertent treatment cannot be ruled out.
- The results of this systematic review and other evidence sources show important differences in the epidemiology and dynamics of *M. genitalium* and *Chlamydia trachomatis* infection.
