# Supplementary material for: Mycoplasma genitalium incidence, persistence, concordance between partners and progression: systematic review and meta-analysis
Source: Sex Transm Infect. 2019 May 4;95(5):328–35. doi: 10.1136/sextrans-2018-053823 (PMC6678058; doi:10.1136/sextrans-2018-053823)
Supplement: Supplementary data [file sextrans-2018-053823supp002.pdf]

## Online supplementary file 2

# *Mycoplasma genitalium* incidence, persistence, concordance between partners and progression: systematic review and meta-analysis

Manuel Cina,[1] Lukas Baumann,[1] Dianne Egli-Gany,[1] Florian S Halbeisen,[1] Hammad Ali,[2] Pippa Scott,[1,3] Nicola Low[1]

1. Institute of Social and Preventive Medicine, University of Bern, Bern, Switzerland;
2. Kirby Institute, University of New South Wales, Sydney, Australia;
3. Department of Pathology and Biomedical Science, University of Otago, Christchurch, New Zealand

# Online supplementary file 2

## Contents

Supplementary text, Text S1 to Text S4 .................................................... 3
Text S1. Medline search strategy .................................................... 3
Text S2. Embase search strategy .................................................... 4
Text S3. Additional databases (IndMED, LILACS, African Index Medicus) ..................... 5
Text S4. Assessment of risk of bias and reporting .................................................... 5

Supplementary figures and tables, Figure S1 and Figure S2, Table S1 to Table S11 6

.................................................... 6
Figure S1. Flow Chart .................................................... 6
Table S1. List of included studies with study name, references, and linked references from systematic review of *M. genitalium* prevalence .................................................... 7
Table S2. Included studies, incidence of *M. genitalium*, by study name .......................... 10
Table S3. Risk of bias assessment, studies reporting incidence of *M. genitalium*, by study name .................................................... 11
Table S4. Included studies, duration of persistent detection of *M. genitalium*, by study name .................................................... 12
Figure S2. Persistent detection of *M. genitalium*, shown as proportion of infected individuals over time. Each study started with 100% infected individuals at baseline. ...... 13
Table S5. Risk of bias assessment, studies reporting duration of persistent detection of *M. genitalium*, by study name .................................................... 14
Table S6. Included studies, concordance of *M. genitalium* status between sexual partners, by study type and study name .................................................... 15
Table S7. Risk of bias assessment, concordance of *M. genitalium* status, by study type and study name .................................................... 17
Table S8. Test of interaction for study design concordance studies................................ 18
Table S9. Included studies, progression to PID, by study type and study name .............. 19
Table S10. Risk of bias assessment, prospective studies reporting progression to PID, by study type and study name .................................................... 20
Table S11. Infection parameters for *M. genitalium* and *C. trachomatis* in studies with data about both infections .................................................... 21

# Online supplementary file 2

# Supplementary text, Text S1 to Text S4

## Text S1. Medline search strategy

1: „Mycoplasma genitalium"[Mesh]
2: Mycoplasma genitalium
3: 1 OR 2

4: "Mycoplasma Infections"[Mesh]
5: Mycoplasma
6: Mycoplasm*
7: 4 OR 5 OR 6

8: "Reproductive Tract Infections"[Mesh]
9: genital tract
10: reproductive tract

11: "Salpingitis"[Mesh]
12: Salpingitis
13: "Endometritis"[Mesh]
14: Endometritis
15: "Parametritis"[Mesh]
16: Parametritis
17: "Oophoritis"[Mesh]
18: Oophoritis
19: Ovary
20: Metritis
21: Pelviperitonitis

22: "Pelvic Inflammatory Disease"[Mesh]
23: p.i.d.
24: pelvis
25: pelvic
26: Adnexitis

27: "Sexually Transmitted Diseases"[Mesh]
28: sexually transmitted
29: STD
30: STDs
31: VD
32: Sexual disease transmission
33: Veneral
34: Venereal

35: Genital*
36: Vagina*
37: Endometri*
38: Cervix
39: Cervical*
40: Urethra*
41: Fallopian
42: tuba*
43: tube
44: tubes
45: 8 OR 9 OR 10 OR 11 OR 12 OR 13 OR 14 OR 15 OR 16 OR 17 OR 18 OR 19 OR 20 OR 21 OR 22 OR 23 OR 24 OR 25 OR 26 OR 27 OR 28 OR 29 OR 30 OR 31 OR 32 OR 33 OR 34 OR 35 OR 36 OR 37 OR 38 OR 39 OR 40 OR 41 OR 42 OR 43 OR 44
46: 3 OR (7 AND 45)

Filters: 1981/01/01 – 2018/17/03
Humans

# Online supplementary file 2

## Text S2. Embase search strategy

1: 'mycoplasma genitalium'/exp
2: Mycoplasma genitalium
3: 'mycoplasma genitalium'
4: 1 OR 2 OR 3

5: 'mycoplasmosis'/exp
6: 'mycoplasma'/exp
7: 'mycoplasma'
8: mycoplasm*
9: 5 OR 6 OR 7 OR 8

10: 'genital tract infection'/exp
11: genital tract
12: reproductive tract

13: 'adnexitis'/exp
14: adnexitis
15: ‚metritis'/exp
16: Metritis
17: 'endometritis'/exp
18: Endometritis
19: Parametritis
20: ‚ovary inflammation'/exp
21: Ovary
22: 'pelviperitonitis'/exp
23: pelviperitonitis
24: 'pelvis abscess'/exp
25: pelvis abscess
26: 'salpingitis'/exp
27: salpingitis

28: 'pelvic inflammatory disease'/exp
29: p.i.d.
30: pelvic
31: pelvis

32: sexually transmitted
33: sexual disease transmission
34: std
35: stds
36: vd
37: veneral
38: venereal

39: Genital*
40: Vagina*
41: Endometri*
42: Cervi*
43: Urethra*
44: Fallopian
45: tuba*
46: tube
47: tubes
48: 10 OR 11 OR 12 OR 13 OR 14 OR 15 OR 16 OR 17 OR 18 OR 19 OR 20 OR 21 OR 22 OR 23 OR 24 OR 25 OR 26 OR 27 OR 28 OR 29 OR 30 OR 31 OR 32 OR 33 OR 34 OR 35 OR 36 OR 37 OR 38 OR 39 OR 40 OR 41 OR 42 OR 43 OR 44 OR 45 OR 46 OR 47

49: 4 OR (9 AND 48)

# Online supplementary file 2

## Text S3. Additional databases (IndMED, LILACS, African Index Medicus)

„*Mycoplasma genitalium*“

Abbreviations: IndMED, Indian Medical Journals; LILACS Latin American and Carribean Health Sciences Literature

## Text S4. Assessment of risk of bias and reporting

We based our assessment on two tools. We first applied a tool based on a tool for evaluating prevalence studies [1] because we had used this tool in a systematic review of prevalence studies of chlamydia infection [2] and in our linked systematic review of prevalence of *M. genitalium* [3]. We used this tool for cross-sectional studies of concordance. The tool includes elements of both risk of bias and reporting. We used the items about reporting of confidence intervals and raw data for all study designs [1]. We used the classification of responses (‘yes’, ‘no’, ‘unclear/not reported’) in both assessment tools.

For cohort studies and nested case-control studies, which were the study designs used for incidence and persistent detection of *M. genitalium* and risk of progression from lower genital tract *M. genitalium* to PID, we used relevant items from the Cochrane Collaboration Methods Group Tool to Assess Risk of Bias in Cohort Studies [4]. For consistency across study designs, we used the items about the source population from the tool for evaluating prevalence studies to assess selection of exposed and non-exposed cohorts (item 1). We did not assess matching on prognostic variables (item 4) but we used item 5 (assessment of presence or absence of prognostic factors). We only assessed co-interventions (item 8) for studies of PID.

**References**


1. Boyle MH. Guidelines for evaluating prevalence studies. *Evidence-Based Mental Health* 1998;1(2):37-40.

2. Redmond SM, Alexander-Kisslig K, Woodhall SC, et al. Genital chlamydia prevalence in Europe and non-European high income countries: systematic review and meta-analysis. *PLoS One* 2015;10(1):e0115753.

3. Baumann L, Cina M, Egli-Gany D, et al. Prevalence of Mycoplasma genitalium in different population groups: systematic review andmeta-analysis. *Sex Transm Infect* 2018;94(4):255-62.

4. Cochrane Collaboration. Tool to Assess Risk of Bias in Cohort Studies. <https://methods.cochrane.org/bias/sites/methods.cochrane.org.bias/files/public/uploads/Tool%20to%20Assess%20Risk%20of%20Bias%20in%20Cohort%20Studies.pdf>, (accessed 20 December 2018.).

# Online supplementary file 2

## Supplementary figures and tables, Figure S1 and Figure S2, Table S1 to Table S11

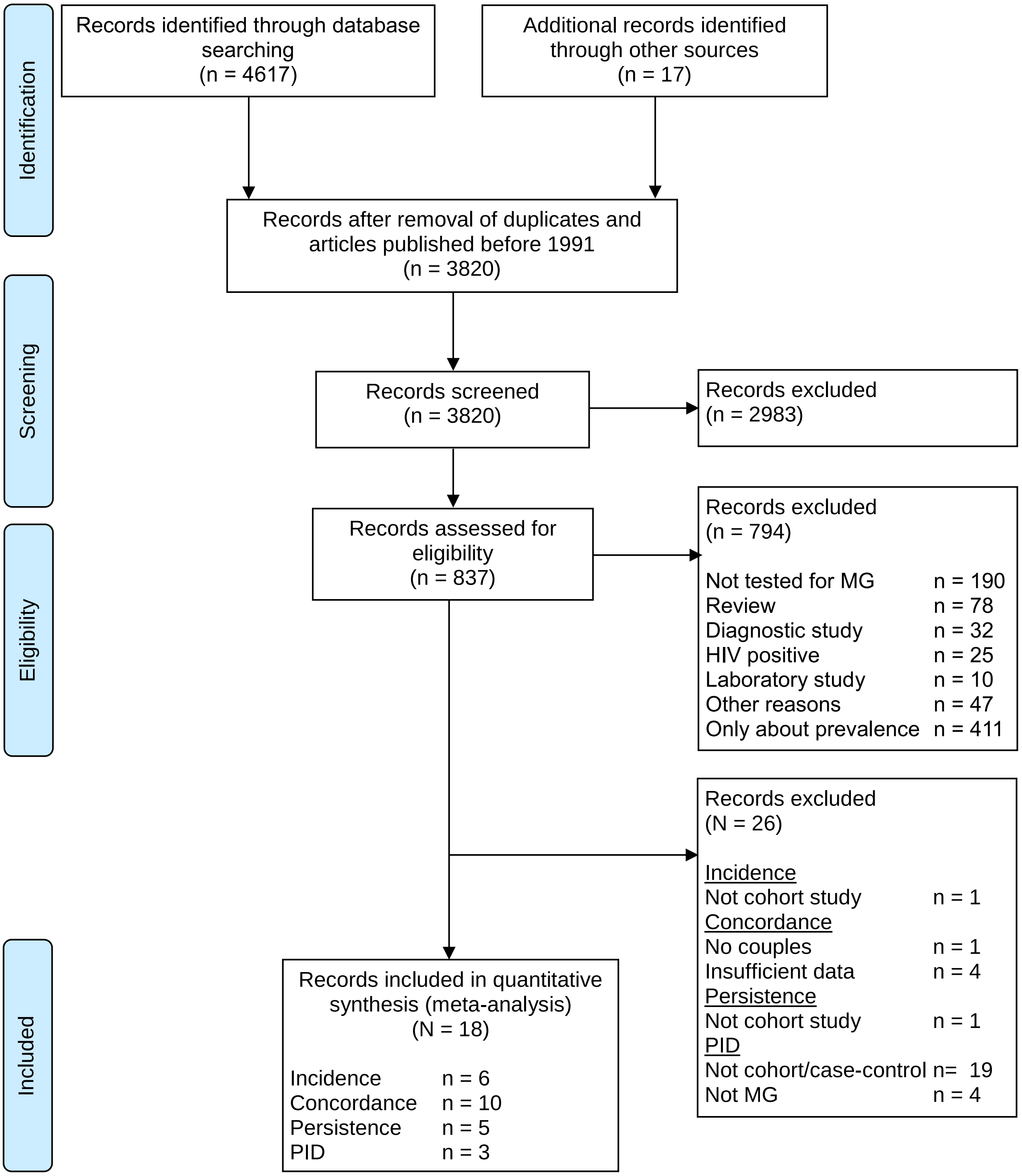


Figure S1. Flow Chart

Abbreviations: MG, *Mycoplasma genitalium*; HIV, human immunodeficiency virus; PID, pelvic inflammatory disease

## Online supplementary file 2

Table S1. List of included studies with study name, references, and linked references from systematic review of *M. genitalium* prevalence

| **Study identifier** | **References** | **Linked reference from review of *M. genitalium* prevalence**[a] |
|---|---|---|
| Australia 3 [29] | Walker J, Fairley CK, Bradshaw CS, et al. Mycoplasma genitalium incidence, organism load, and treatment failure in a cohort of young Australian women. Clin Infect Dis 2013;56(8):1094-100. | Walker J, Fairley CK, Bradshaw CS, et al. The difference in determinants of Chlamydia trachomatis and Mycoplasma genitalium in a sample of young Australian women. BMC Infect Dis 2011;11:35. |
| Australia 6 [40] | Slifirski JB, Vodstrcil LA, Fairley CK, et al. Mycoplasma genitalium Infection in adults reporting sexual contact with infected partners, Australia, 2008-2016. Emerging Infectious Diseases 2017;23(11):1826-33. | |
| Great Britain 2 [5] | Oakeshott P, Aghaizu A, Hay P, et al. Is Mycoplasma genitalium in women the "New Chlamydia?" A community-based prospective cohort study. Clin Infect Dis 2010;51(10):1160-6. | Same |
| Great Britain 8 [34] | Keane FE, Thomas BJ, Gilroy CB, et al. The association of Mycoplasma hominis, Ureaplasma urealyticum and Mycoplasma genitalium with bacterial vaginosis: observations on heterosexual women and their male partners. Int J STD AIDS 2000;11(6):356-60. | |
| Great Britain 9 [33] | Keane FE, Thomas BJ, Gilroy CB, et al. The association of Chlamydia trachomatis and Mycoplasma genitalium with non-gonococcal urethritis: observations on heterosexual men and their female partners. Int J STD AIDS 2000;11(7):435-9. | |
| Kenya 2 [26] | Cohen CR, Nosek M, Meier A, et al. Mycoplasma genitalium infection and persistence in a cohort of female sex workers in Nairobi, Kenya. Sex Transm Dis 2007;34(5):274-9. | |
| Kenya 3 [28] | Lokken EM, Balkus JE, Kiarie J, et al. Association of recent bacterial vaginosis with acquisition of Mycoplasma genitalium. Am J Epidemiol 2017;186(2):194-201. | |

# Online supplementary file 2

| Study identifier | References | Linked reference from review of *M. genitalium* prevalence[a] |
|---|---|---|
| Peru 1 [35] | Nelson A, Press N, Bautista CT, et al. Prevalence of sexually transmitted infections and high-risk sexual behaviors in heterosexual couples attending sexually transmitted disease clinics in Peru. Sex Transm Dis 2007;34(6):344-61. | |
| Sweden 2 [36] | Falk L, Fredlund H, Jensen JS. Symptomatic urethritis is more prevalent in men infected with Mycoplasma genitalium than with Chlamydia trachomatis. Sex Transm Infect 2004;80(4):289-93. | Same |
| Sweden 5 [37] | Anagrius C, Lore B, Jensen JS. Mycoplasma genitalium: prevalence, clinical significance, and transmission. Sex Transm Infect 2005;81(6):458-62. | Same |
| Sweden 10 [41] | Bjartling C, Osser S, Persson K. The association between Mycoplasma genitalium and pelvic inflammatory disease after termination of pregnancy. BJOG 2010;117(3):361-4. | |
| Sweden 11 [38] | Falk L, Fredlund H, Jensen JS. Signs and symptoms of urethritis and cervicitis among women with or without Mycoplasma genitalium or Chlamydia trachomatis infection. Sex Transm Infect 2005;81(1):73-8. | |
| Sweden 12 [39] | Wikstrom A, Jensen JS. Mycoplasma genitalium: a common cause of persistent urethritis among men treated with doxycycline. Sex Transm Infect 2006;82(4):276-9. | |
| USA/Kenya 1 [30] | Balkus JE, Manhart LE, Lee J, et al. Periodic presumptive treatment for vaginal infections may reduce the incidence of sexually transmitted bacterial infections. J Infect Dis 2016; 213: 1932-7 | |
| Uganda 1 [27] | Vandepitte J, Weiss HA, Kyakuwa N, et al. Natural history of Mycoplasma genitalium infection in a cohort of female sex workers in Kampala, Uganda. Sex Transm Dis 2013;40(5):422-7. | Vandepitte J, Muller E, Bukenya J, et al. Prevalence and correlates of Mycoplasma genitalium infection among female sex workers in Kampala, Uganda. J Infect Dis 2012;205(2):289-96. |

# Online supplementary file 2

| Study identifier | References | Linked reference from review of ***M. genitalium*** **prevalence**[a] |
|---|---|---|
| USA 6 [42] | Haggerty CL, Totten PA, Astete SG, et al. Failure of cefoxitin and doxycycline to eradicate endometrial Mycoplasma genitalium and the consequence for clinical cure of pelvic inflammatory disease. Sex Transm Infect 2008;84(5):338-42. | |
| USA 7 [31] | Tosh AK, Van Der Pol B, Fortenberry JD, et al. Mycoplasma genitalium among adolescent women and their partners. J Adolesc Health 2007;40(5):412-7. | |
| USA 8 [32] | Thurman AR, Musatovova O, Perdue S, et al. Mycoplasma genitalium symptoms, concordance and treatment in high-risk sexual dyads. Int J STD AIDS 2010;21(3):177-83. | |

[a] Baumann L, Cina M, Egli-Gany D, et al. Prevalence of *Mycoplasma genitalium* in different population groups: systematic review and meta-analysis. Sex Transm Infect 2018;94:255-62.

# Online supplementary file 2

Table S2. Included studies, incidence of *M. genitalium*, by study name

| Study identifier | First author | Year | Type of specimen collected for analysis | Infectious state of participants at start of study | Number of participants at baseline | Frequency of follow-up | Duration of follow-up | Reported outcome |
|---|---|---|---|---|---|---|---|---|
| Australia 3 [29] | Walker J | 2013 | Vaginal swab | Uninfected and infected participants without treatment | 1110 | Every six months | 12 months | 1.3 incident infections per 100 person-years (95% CI, .8, 2.3) |
| Great Britain 2 [5] | Oakeshott P | 2010 | Vaginal swab | Uninfected participants | 2300 | One follow up | Median 16 months | 0.91% incident infections per year (95% CI, 0.46%, 1.63%) |
| Kenya 2 [26] | Cohen CR | 2007 | Cervical swab and endometrial biopsy | Uninfected and infected participants without treatment | 299 | Every two months | Up to 33 months | 22.7 incident infections per 100 women-years |
| Kenya 3 [28] | Lokken EM | 2017 | Vaginal swab | Uninfected and infected participants without treatment | 280 | Every month | Not reported | 34.6 incident infections per 100 person-years (95% CI, 26, 42) |
| Uganda 1 [27] | Vandepitte J | 2013 | Cervical swab | Uninfected participants | 111 | Every three months | 12 months | 6.6 recurrent infections per 100 person-years (95% CI, 4.8, 9.0) |
| USA/Kenya 1 [30] | Balkus JE | 2016 | Genital swab | Uninfected participants | 101 | Every two months | 12 months | 40.3 incident infections per 100 person-years (95% CI, 28.5, 56.9) |

Abbreviations: CI, confidence interval

## Online supplementary file 2

Table S3. Risk of bias assessment, studies reporting incidence of *M. genitalium*, by study name

| Study | Target population clearly defined? (yes, no) | Source population clearly defined? (yes, no) | Source population adequate sample of target population? (yes, no) | Can we be confident in the assessment of the exposure? (yes, no) | Can we be confident in the assessment of incident infections? (yes, no) | Can we be confident in the assessment of the presence or absence of prognostic factors? (yes, no) | Can we be confident that individuals lost to follow-up are similar to individuals followed-up until end of study? (yes, no, unclear/not reported) | Proportion of participants lost to follow-up adequate? (< 20%, 20-30%, > 30%, unclear/not reported) | Confidence intervals included for incidence? (yes, no) | Data provided to calculate reported result? (yes, no) | Data provided to calculate 95% CI for reported result? (yes, no) |
|---|---|---|---|---|---|---|---|---|---|---|---|
| Australia 3 [29] | | | | | | | | | | | |
| Great Britain 2 [5] | | | | | | | | | | | |
| Kenya 2 [26] | | | | | | | | | | | |
| Kenya 3 [28] | | | | | | | | | | | |
| Uganda 1 [27] | | | | | | | | | | | |
| USA/Kenya 1 [30] | | | | | | | | | | | |

Adapted from: Cochrane Collaboration. Tool to Assess Risk of Bias in Cohort Studies. <http://methods.cochrane.org/bias/sites/methods.cochrane.org.bias/files/public/uploads/Tool%20to%20Assess%20Risk%20of%20Bias%20in%20Cohort%20Studies.pdf >, (accessed 21 December 2018.)

## Online supplementary file 2

Table S4. Included studies, duration of persistent detection of *M. genitalium*, by study name

| Study identifier | First Author | Year | Type of specimen collected for analysis | Number of participants at baseline | Duration of follow-up | Reported information about persistence | Efforts made to differ persistent infections from reinfections | Antibiotic treatment for other STI |
|---|---|---|---|---|---|---|---|---|
| Great Britain 2 [5] | Oakeshott P | 2010 | Vaginal swab | 78 | Median 16 months | 7 of 27 women had persistent positive samples after 12-27 months | Genotyping | Treatment (not further specified) for *C. trachomatis* at baseline in intervention arm of the clinical trial. Possible testing and treatment before follow-up. |
| Kenya 2 [26] | Cohen CR | 2007 | Cervical swab and endometrial biopsy | 107 | Up to 33 months | 56 (52%), 18 (17%), 10 (9%), and 23 (21%) *M. genitalium* infections persisted for 1, 3, 5, and 7 months | Genotyping in 7 selected women persistently infected for 10 months or more | Visits every two months. Doxycycline or ciprofloxacin if infected with *C. trachomatis* or *N. gonorrhoeae* within four days after diagnosis |
| Kenya 3 [28] | Lokken EM | 2017 | Vaginal swab | 280 | 170.5 person years at risk | 18, 7 and 3 *M. genitalium* infections persisted after 100, 200 and 300 days | Not reported | Monthly visits. Immediate syndromic treatment or treatment after diagnosis for sexually transmitted infections according to Kenyan national guidelines. |
| Uganda 1 [27] | Vandepitte J | 2013 | Cervical swab | 148 | 12 months | Only graphical presentation | Not reported | Visits every three months. Treatment for vaginal discharge syndrome with doxycycline, ciprofloxacin, metronidazole. Immediate syndromic treatment or as soon as laboratory results available. |
| USA 7 [31] | Tosh AK | 2007 | Women: Vaginal specimen | 23 | 12 weeks after positive sample | 31.3% of the infections lasted >8weeks<br>21.9% of the infections lasted >12weeks | Short test intervals (weekly) | Treatment for *N. gonorrhoeae, C. trachomatis, T. vaginalis*, candidiasis or bacterial vaginosis according to CDC guidelines within two weeks of diagnosis after every three months. |

Abbreviations: CDC, Centers for Disease Control and Prevention; STI, sexually transmitted infection; PID, pelvic inflammatory disease

# Online supplementary file 2

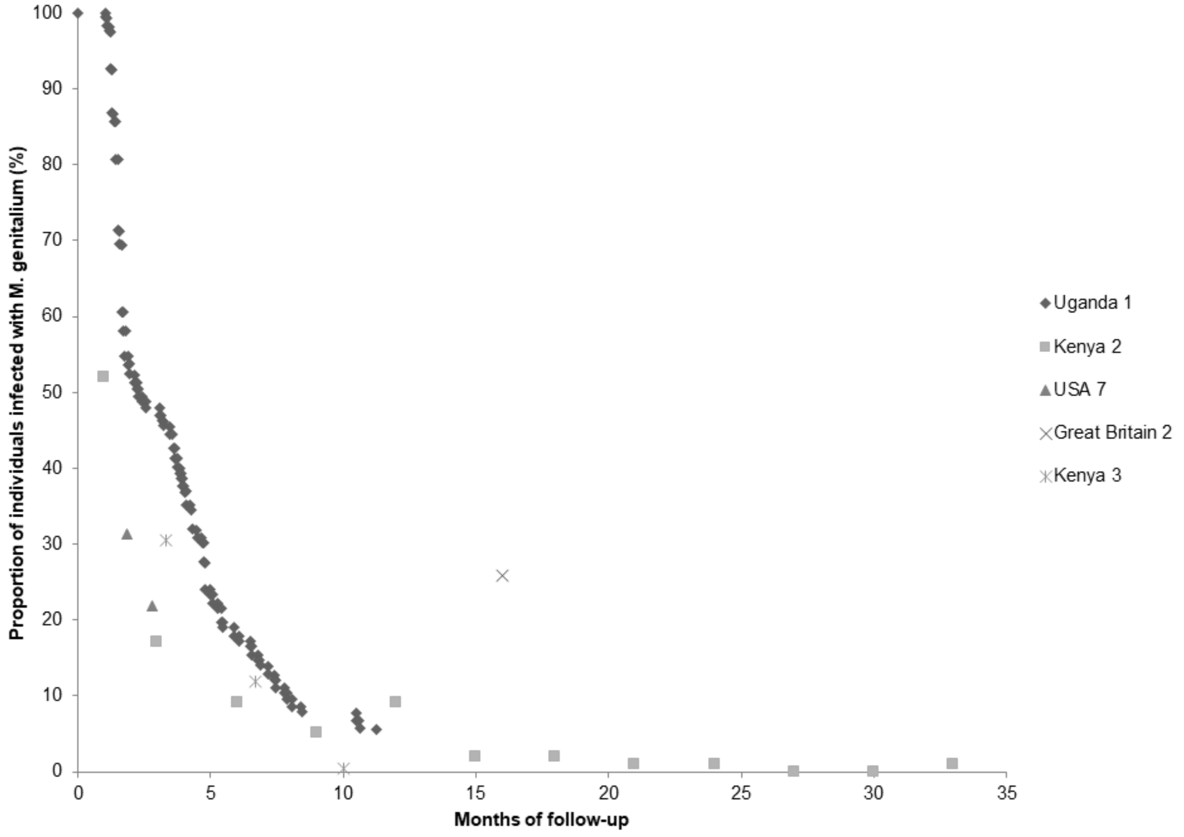


Figure S2. Persistent detection of *M. genitalium*, shown as proportion of infected individuals over time. Each study started with 100% infected individuals at baseline.

# Online supplementary file 2

Table S5. Risk of bias assessment, studies reporting duration of persistent detection of *M. genitalium*, by study name

| Study | Target population clearly defined? (yes, no) | Source population clearly defined? (yes, no) | Source population adequate sample of target population? (yes, no) | Can we be confident in the assessment of exposure? (yes, no) | Can we be confident in the assessment of the presence or absence of prognostic factors? (yes, no) | Can we be confident in the assessment of outcome? (yes, no) | Can we be confident that individuals lost to follow-up are similar to individuals followed-up until end of study? (yes, no, unclear/not reported) | Proportion of participants lost to follow-up adequate? (< 20%, 20-30%, > 30%, unclear/not reported) | Confidence intervals for reported result included? (yes, no) | Data provided to calculate reported result? (yes, no) |
|---|---|---|---|---|---|---|---|---|---|---|
| Great Britain 2 [5] | | | | | | | | | | |
| Kenya 2 [26] | | | | | | | | | | |
| Kenya 3 [28] | | | | | | | | | | |
| Uganda 1 [27] | | | | | | | | | | |
| USA 7 [31] | | | | | | | | | | |

Adapted from: Cochrane Collaboration. Tool to Assess Risk of Bias in Cohort Studies. <http://methods.cochrane.org/bias/sites/methods.cochrane.org.bias/files/public/uploads/Tool%20to%20Assess%20Risk%20of%20Bias%20in%20Cohort%20Studies.pdf> (accessed 21 December 2018).

# Online supplementary file 2

Table S6. Included studies, concordance of *M. genitalium* status between sexual partners, by study type and study name

| Study identifier | First author | Year | Study design | Type of specimen collected for analysis | Enrolment procedure | Number of couples/number of index cases |
|---|---|---|---|---|---|---|
| Great Britain 8 [34] | Keane FEA | 2000 | Partner study | Women: Vaginal and endocervical sample<br>Men: Urine sample | Participants approached in STI clinics and asked to invite their current sexual partner if he/she was not yet present | 34 |
| Great Britain 9 [33] | Keane FEA | 2000 | Partner study | Women: Vaginal and endocervical sample<br>Men: Urine sample | Participants approached in STI clinics and asked to invite their current sexual partner if he/she was not yet present | 29 |
| Peru 1 [35] | Nelson A | 2007 | Partner study | Women: Clean-catch urine, pharyngeal, rectal, vaginal, endocervical swab<br>Men: Clean-catch urine, pharyngeal, rectal, urethral swab | Participants approached in STI clinics and asked to invite their current sexual partner if he/she was not yet present | 195 |
| USA 7 [31] | Tosh AK | 2007 | Partner study | Women: Vaginal specimen<br>Men: Urine sample | Recent sexual partners of participants approached by telephone calls or field visits | 117 |
| USA 8 [32] | Thurman AR | 2010 | Partner study | Women: Urine and endocervical sample<br>Men: Urine sample | STI clinic attendees with a positive test for a non *M. genitalium* STI were asked to bring their current sexual partner to a research clinic | 494 |
| Australia 6 [40] | Slifirski JB | 2017 | Index cases | Women: mostly vaginal or endocervical samples; anorectal sample if anal sex<br>Men: mostly first void urine; anorectal sample if anal sex | People attending as contacts of a person with *M. genitalium* and lab result of the positive index case | 377 |
| Sweden 2 [36] | Falk L | 2004 | Index cases | First void urine | Current sexual partners of *M. genitalium* positive index cases asked to come to the clinic by their partner or by partner notification | 18 |
| Sweden 5 [37] | Anagrius C | 2005 | Index cases | Women: Endocervical sample, placed in tube with fist void urine<br>Men: First void urine | Current sexual partners of *M. genitalium* positive index cases asked to come to the clinic by their partner or by partner notification | 52 |

# Online supplementary file 2

| | | | | | | |
|---|---|---|---|---|---|---|
| Sweden 11 [38] | Falk L | 2005 | Index cases | Women: First void urine and endocervical sample<br>Men: First void urine | Current sexual partners of *M. genitalium* positive index cases asked to come to the clinic by their partner or by partner notification | 21 |
| Sweden 12 [39] | Wikstrom A | 2006 | Index cases | First void urine | Current sexual partners of *M. genitalium* positive index cases asked to come to the clinic by their partner or by partner notification | 9 |

Abbreviations: STI, sexually transmitted infection

# Online supplementary file 2

Table S7. Risk of bias assessment, concordance of *M. genitalium* status, by study type and study name

| Study | Target population clearly defined? (yes, no) | Source population clearly defined? (yes, no) | Source population adequate sample of target population? (yes, no) | Response rate at baseline (>80%, 70-80%, > 70%, unclear/not reported) | Similar socio-demographic attributes of responders and non-responders? (yes, no, unclear/not reported) | Was a probability sampling used? (yes, no, unclear/not reported) | Was a sample size calculation reported? (yes, no, unclear/not reported) | Was the achieved sample size at least as good as in the sample size calculation? (yes, no, unclear/not reported) | Can we be confident in the assessment of exposure? (yes, no) | All data about infectious state of couples provided? (yes, no) |
|---|---|---|---|---|---|---|---|---|---|---|
| Great Britain 8 [34] | [red] | [green] | [red] | [red] | | [red] | | | [green] | [green] |
| Great Britain 9 [33] | [red] | [green] | [red] | | | [red] | | | [green] | [red] |
| Peru 1 [35] | [green] | [green] | [red] | [red] | | [red] | | | [green] | [red] |
| USA 7 [31] | [green] | [green] | [green] | | | [red] | | | [green] | [red] |
| USA 8 [32] | [green] | [red] | [red] | | | [red] | | | [green] | [green] |
| Australia 6 [40] | [green] | [green] | [green] | | | [red] | | | [red] | [red] |
| Sweden 2 [36] | [green] | [green] | [green] | | | [red] | | | [green] | [green] |
| Sweden 5 [37] | [green] | [green] | [green] | | | [red] | | | [red] | [green] |
| Sweden 11 [38] | [green] | [green] | [green] | | | [red] | | | [green] | [green] |
| Sweden 12 [39] | [green] | [green] | [green] | | | [red] | | | [green] | [green] |

Adapted from: Redmond SM, Alexander-Kisslig K, Woodhall SC, et al. Genital chlamydia prevalence in Europe and non-European high income countries: systematic review and meta-analysis. *PLoS One* 2015; **10**(1): e0115753.

# Online supplementary file 2

Table S8. Test of interaction for study design concordance studies

| Sex of index cases | Average difference in mean between index case studies and partner studies | Standard error | 95% CI difference | t | P>t | Residual $I^2$, % |
|---|---|---|---|---|---|---|
| Male | 0.117 | 0.083 | -0.313 to 0.313 | 1.42 | 0.198 | 21.7 |
| Female | 0.023 | 0.145 | -0.332 to 0.377 | 0.16 | 0.881 | 67.0 |

# Online supplementary file 2

Table S9. Included studies, progression to PID, by study type and study name

| Study identifier | First author | Year | Study type | Sampling method | Type of specimen collected for analysis | Diagnosis of PID | Number of participants at baseline | Duration of follow-up | Result |
|---|---|---|---|---|---|---|---|---|---|
| Great Britain 2 [5] | Oakeshott P | 2010 | Cohort study | Convenience sample | Vaginal swab | CDC Guidelines 2010. Hager's criteria. Women were also categorized as having PID if a health care professional had treated them for PID. | 2378 | 12 months | PID in *M. genitalium* positives: 3.9%<br>PID in *M. genitalium* negatives: 1.7%<br>Absolute risk increase: 2.2% |
| USA 6 [42] | Haggerty CL | 2008 | Cohort study | Convenience sample | Cervical swab & endometrial biopsy | At least five neutrophils in the endometrial surface epithelium in the absence of menstrual endometrium and/or at least two plasma cells in the endometrial stroma in the endometrial biopsy. | 682 | 30 days | PID in *M. genitalium* positives: 6.8%<br>PID in *M. genitalium* negatives: 7%<br>Absolute risk increase: 0.2% |
| Sweden 10 [41] | Bjartling C | 2010 | Nested case-control study | Convenience sample | Urine & cervical or vaginal swab | CDC Guidelines 2010 | 2079 | 6 weeks | *M. genitalium* positive with PID: 12.2%<br>*M. genitalium* negative with PID: 2.4% |

Abbreviations: PID, pelvic inflammatory disease; CDC, Centers for Disease Control and Prevention

CDC Guidelines 2010 for the diagnosis of PID/Modified Hager's criteria: No cause for the illness other than PID identified and one or more of cervical motion tenderness, uterine tenderness and adnexal tenderness present on pelvic examination. Diagnosis made more specific by one or more of: oral temperature >101° F (>38.3° C), abnormal cervical or vaginal mucopurulent discharge, presence of abundant numbers of WBC on saline microscopy of vaginal fluid, elevated erythrocyte sedimentation rate, elevated C-reactive protein and laboratory documentation of cervical infection with *N. gonorrhoeae* or *C. trachomatis.*

# Online supplementary file 2

Table S10. Risk of bias assessment, prospective studies reporting progression to PID, by study type and study name

| Study | Can we be confident in the assessment of the presence or absence of prognostic factors? (yes, no) | Can we be confident in the assessment of outcome? (yes, no) | Can we be confident that individuals lost to follow-up are similar to individuals followed-up until end of study? (yes, no, unclear/not reported) | Proportion of participants lost to follow-up adequate? (< 20%, 20-30%, > 30%, unclear/not reported) | Can we be confident that co-interventions were similar between groups? (e.g. antibiotic treatment?) (yes, no) | Confidence intervals included for the association of PID with M. genitalium? (yes, no) | Data provided to calculate the odds/risk ratio for the association of PID with M. genitalium? (yes, no) |
|---|---|---|---|---|---|---|---|
| Great Britain 2 [5] | | | | | | | |
| USA 6 [40] | | | | | | | |
| Sweden 10 [41] | | | | | | | |

Abbreviations: PID, pelvic inflammatory disease

Cochrane Collaboration. Tool to Assess Risk of Bias in Cohort Studies. <http://methods.cochrane.org/bias/sites/methods.cochrane.org.bias/files/public/uploads/Tool%20to%20Assess%20Risk%20of%20Bias%20in%20Cohort%20Studies.pdf>, (accessed 21 December 2018).

## Online supplementary file 2

Table S11. Infection parameters for *M. genitalium* and *C. trachomatis* in studies with data about both infections

| Study ID | Study population | Infection | Prevalence, % (95% CI) | Incidence, per 100 person-years (95% CI) | Persistence of untreated infection, from study, median years | Persistence as prevalence/ incidence, years | References |
|---|---|---|---|---|---|---|---|
| Australia 3 [29] | Young women aged 16-25 years; primary health clinics in Australia | MG | 2.4 (1.5, 3.3) | 1.3 (0.7, 2.2) | Not measured | 1.85 | MG, CT prevalence: Walker J, Fairley CK, Bradshaw CS, et al. BMC Infect Dis 2011;11:35. |
| | | CT | 4.9 (2.9, 7.0) | 4.4 (3.3, 5.9) | Not measured | 1.11 | MG incidence: Walker J, Fairley CK, Bradshaw CS, et al. Clin Infect Dis 2013;56(8):1094-100. CT Incidence: Walker J, Tabrizi SN, Fairley CK, et al. PLoS One. 2012;7(5):e37778. |
| Great Britain 2 [5] | Female students aged ≤27 years; London universities and further education colleges, Great Britain | MG | 3.3 (2.6, 4.1) | 0.9 (0.5, 1.6) | 1.33 | 3.66 | MG prevalence, incidence, persistence, CT prevalence: Oakeshott P, Aghaizu A, Hay P, et al. Clin Infect Dis 2010;51(10):1160-6. |
| | | CT | 5.8 | Not measured | Not measured | Could not calculate | |
| Kenya 2 [26] | Female sex workers aged 18-35 years; Kariobangi Nairobi City Council, Nairobi, Kenya | MG | 16 | 22.7 (17.9, 28.3) | 0.083 | 0.7 | MG prevalence, incidence, persistence, CT prevalence, incidence: Cohen CR, Nosek M, Meier A, et al. Sex Transm Dis 2007;34(5):274-9. |
| | | CT | 8 | 14.1 | Not measured | 0.57 | |

| | | | | | | | |
|---|---|---|---|---|---|---|---|
| Kenya 3 [28] | Female sex workers, median age 35.3 years; municipal STI clinic Mombasa, Kenya | MG | 16.1 | 34.6 (26.3, 44.6) | 0.23 | 0.47 | MG prevalence, incidence, persistence: Lokken EM, Balkus JE, Kiarie J, et al. Am J Epidemiol 2017;186(2):194-201. CT prevalence, incidence: Masese L. Baeten JM, Richardson BA, et al. Sex Transm Dis 2013;40(3):221-5. |
| | | CT | 1.9 | 5.0 | Not measured | 0.38 | |
| Uganda 1 [27] | Female sex workers aged 18-40 years; red light areas within southern Kampala, Uganda | MG | 14.0 (12.0, 17.0) | 6.6 (4.7, 9.0) | 0.18 | 2.12 | MG, CT prevalence: Vandepitte J, Muller E, Bukenya J, et al. J Infect Dis 2012;205(2):289-96. |
| | | CT | 9 (7, 11) | Not measured | Not measured | Could not calculate | MG incidence, persistence: Vandepitte J, Weiss HA, Kyakuwa N, et al. Sex Transm Dis 2013;40(5):422-7. |
| USA/Kenya 1 [30] | High-risk women aged 18-45 years; research clinics in Mombasa and Nairobi, Kenya and Birmingham, USA | MG | 8 | 40.3 (28.5, 56.9) | Not measured | 0.2 | Balkus JE, Manhart LE, Lee J et al. J Infect Dis 2016 ; 213 : 1932-7 |
| | | CT | 7 | 15.6 (9.3, 26.4) | Not measured | 0.4 | |
| USA 7 [31] | Women aged 14-17 years and their partners; urban primary health care centres, Indianapolis, USA | MG | 0.8 | Not reported | ≥12 weeks in 21.9% 3 month data collection periods | Could not calculate | MG prevalence, persistence: Tosh AK, Van Der Pol B, Fortenberry JD, et al. J Adolesc Health 2007;40(5):412-7. |
| | | CT | 10.2 | 34 | Not measured | 0.3 | CT prevalence, incidence Batteiger BE, Tu W, Ofner S, et al. J Infect Dis 2010;201(1):42-51. |

Abbreviations: CT, *C. trachomatis*; MG, *M. genitalium*
